# ENVIRONMENTAL EPIGENETICS AND AGING

**DOI:** 10.1093/geroni/igz038.2696

**Published:** 2019-11-08

**Authors:** Andrea Baccarelli

**Affiliations:** Columbia, Mailman School of Public Health, New York, New York, United States

## Abstract

The human epigenome is a flexible, environmental sensitive component of human biology that changes over time. Multiple studies have identified prospective changes in epigenetic marks that indicate that the epigenome ages as we grow older. These changes have been leveraged to create multiple indicators of age that may also predict mortality and age-related disease. There is ongoing research to determine the extent to which age-related epigenomics changes are inherent to cell biology and/or driven by lifestyle and environmental factors. In this presentation, I will review the current evidence derived from human aging studies and potential contributions to human health and disease. I will discuss the source of data, methodological challenges for large human studies, limitations and possible future directions.

